# Insights into Sensing and Biomedical Domains Using Multi-Synthetic Covalent Organic Frameworks

**DOI:** 10.3390/bios16050280

**Published:** 2026-05-11

**Authors:** Hassan Imam Rizvi, Yuchen Qiao, Shilpa Dabas, Peng Ren, Xuemei Yang

**Affiliations:** Shenzhen Key Laboratory of Organic Pollution Prevention and Control, School of Science, Harbin Institute of Technology Shenzhen, Shenzhen 518055, China; 25bf58011@stu.hit.edu.cn (H.I.R.); qiaoyuchen@hit.edu.cn (Y.Q.); shilpa@hit.edu.cn (S.D.)

**Keywords:** covalent organic frameworks, porous materials, sensors, gas, ions, biomedical

## Abstract

Covalent organic frameworks (COFs) are one of the most important crystalline structures, having high porosity, and are mostly composed of lighter elements, such as H, C, N, O, etc., with covalent bonds between them. They are chemically synthesized in a repetitive arrangement and create a highly effective porous surface area that plays a fundamental role in various applications including sensing and biomedical applications. This study offers an overview of COFs in sensing and biomedical applications and provides a detailed overview of various synthesis procedures of COFs. Next, we explore their innovative sensing performances in the cases of various gases, ions and metals. Finally, it is emphasized that the major biomedical applications of COFs have been addressed regarding diseases and treatment strategies. Overall, this review offers an overview of COFs’ capabilities and promising behaviors in enhancing and revolutionizing sensing and biomedical technologies.

## 1. Introduction

The demand for advanced sensing technologies is increasing along with other problems prevailing globally which are prompted by global warming, pathogenic agents and chronic diseases. Analytical chemistry is no longer a laboratory-based process where mass spectrometry (GC-MS), nuclear magnetic resonance (NMR) or other large instruments are commonly used in a centralized manner, and where it already has seen use. It is officially the new frontier where the emphasis is placed on the development of devices like point-of-care (POC) or the point-of-need (PON) devices. The biosensing systems of these new generations are proposed to be small-sized, inexpensive and capable of real-time-sensitive monitoring without compromising the high requirements of detecting limits and their selectivity for clinical/environmental safety [1,2]. The basic design of a biosensor includes a complicated interaction of two components, which are a molecular recognition component as well as a signal transducer [3,4]. The recognition element must have the ability to be lock-and-key selective in isolating an analyte of interest such as heavy metal ions, complex protein biomarkers, wastewater, soil, etc. [5,6]. Meanwhile, the transducer needs to be capable of converting this evasive molecular interaction to some physical signal, e.g., an optical, electrochemical or piezoelectric signal [7,8]. The traditionally tested similar porous materials were zeolites and activated carbons, but because of the absence of structural periodicity and chemical selectivity, these materials would often experience the potential for low signal-to-noise ratios and low response times [9,10].

It was this disadvantage that resulted in the emergence of reticular chemistry, the science of assembling the miniature building blocks known as molecular building blocks in regular, crystalline structures. There are various porous and high-performance structures such as covalent organic frameworks (COFs), Molecular Organic Frameworks (MOFs), Porous Organic Polymers (POPs), etc. MOFs, first described in 1995, contain metal ions or clusters held by organic ligands. By far, MOFs are restricted by the inability to be chemically and thermally stable, although they can approach extremely high surface areas and provide extremely high structural flexibility [11]. These coordination bonds are weak to hydrolysis, thermal instability, and chemical attack (acids and bases), leading to the loss of structure under extreme operating parameters, particularly in polar solvents commonly employed in chemical devices. This is one of the main weaknesses that lead to seeking alternative structures. In comparison, the porous materials known as COFs were first reported more than a decade later in 2005, and include more light elements (H, B, C, N, O), strongly bound by covalent bonds like C-C, C=N and C-O. This important bonding difference allows COFs to be more inherently stable, survive boiling water and high levels of acidity and basicity, and avoid the risk of release of toxic metal ions [12]. Additionally, the all-organic character of COFs results in reduced framework densities and greater flexibility in manipulating the electronic properties through an organic architecture rather than the predominant influence of metal clusters on the electronic properties of MOFs. These properties render COFs good candidates in applications where chemical and thermal stabilities are needed over a long period of time [13,14].

The unique potential of COFs becomes even more conspicuous in comparison to the conventional porous carbons (e.g., activated carbons) and POPs. The porous carbons are very stable and possess large surface areas, yet they are amorphous, and they have large, undefined distributions of pore sizes and thus cannot be molecularly sieved/molecular-size-separated to facilitate catalysis. Synthetic tunable and processable amorphous POPs include hyper-crosslinked polymers but do not have long-range periodicity [15]. This property leads to the lack of a crystalline pore environment with the consequence that it is hard to know structure–function relationships and precise orientations of active sites. COFs possess the chemical stability of a covalent bonding network, and a long-range crystalline order. This enables the COFs to have unimodal pores of homogeneous size, and to spatially regulate the location of functional groups at the atomic level that in turn enable predictable size selectivity and making customized active catalytic or recognition centers [16]. Covalent organic frameworks (COFs) are considered to be one of the brightest examples of emerging products in this area [17,18,19]. Because of their first application in 2005, COFs have changed the limits of organic material science [20,21,22,23]. Informally called COFs, they are all-light-center (C, H, O, N, B) frameworks made by self-assembly. They are characterized by the crystalline structure, either 2D or 3D, which is the result of the reversibility of dynamic covalent chemistry [24,25,26,27]. This reversibility allows error correction in the synthesis to produce highly ordered, polygonal systems of pores which are unparalleled in structural accuracy. COFs are perhaps most useful in biosensing due to the fact that, in this respect, this approach of architectural programmability is their key strength [28,29,30,31,32]. Researchers are able to pre-program the physical and chemical environment of the structure of the framework by carefully choosing the geometry and functionality of the organic monomers, the “knots and the edges” [33,34]. As an example, imine, hydrazone or keto-enamine cleavage can be introduced to give the sensor extraordinary chemical stability and capability to work under the extreme acidic or alkaline conditions that are typical of biological fluids [35,36,37]. Furthermore, due to the excellent porosity and internal surface area, COFs exhibit rapid mass transport of the analytes because of their permanent porosity’s ability to surmount the diffusion limitations that plague non-porous sensing films [38,39,40].

**Figure 1:**
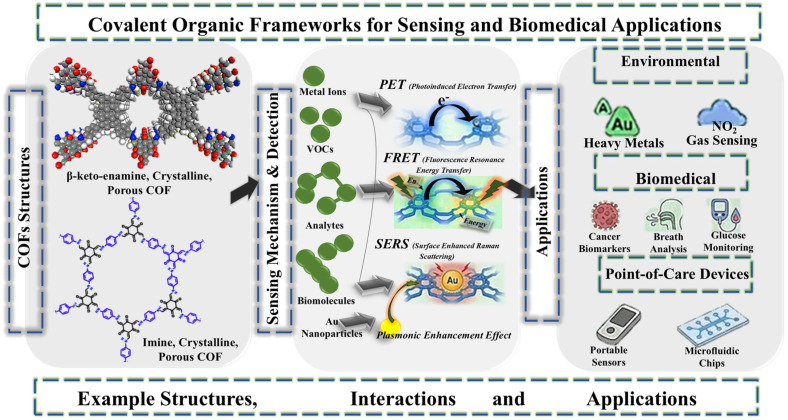
General overview of COFs for sensing and biomedical applications. Reproduced with permission from ref. [60,61]. Copyright 2020, American Chemical Society.

COFs can be universally post-synthetically modified in addition to structural benefits that are already present. The regular organization of pores allows the particular stabilization of second messengers (i.e., aptamers, enzymes, fluorophores or gold nanoparticles) [41,42,43]. This hybridization capability makes it possible for one COF platform to have numerous functions at once: it can also act as a sieve to reject interferents, a concentrator to enrich the analyte, and a signal amplifier to decrease the limit of detection. In the biomedical world, discoveries have been made in terms of the detection of circulating tumor cells; monitoring glucose, since it was first used in the treatment of diabetes; and the detection of volatile organic compounds (VOCs) in human breath [44,45,46,47]. The general overview of imine-based COFs and their sensing as well as biomedical applications is presented in Figure 1. With the arrival of intelligent materials, the functions of COFs are growing beyond being passive adsorbers to being active participants in sensing mechanisms [48,49]. Also, common to the high-technology biosensors developed on the principles of COFs is the use of advanced processes such as photoinduced electron transfer (PET), Förster resonance energy transfer (FRET) and surface-enhanced Raman scattering (SERS) [50,51,52,53]. The analyte can be detected at the picomolar or even smaller level using these mechanisms, which is the level of sensitivity needed in the detection of the disease at an early stage in cases when biomarkers can be detected in minute quantities. In PET systems, the transfer of electrons between a fluorophore and an adjacent unit of donor or acceptor takes place, regulating the fluorescence intensity contingent on the binding of the analyte. The working of the mechanism is that the electron transfer pathway quenches the emission of the fluorophore, the pathway is blocked and the fluorescence turns on. COF-PET sensors usually have a detection limit in the low-nanomolar range for pollutants of the environment (nitroaromatic compounds and heavy metal ions) [54]. An example is the recent COF-based PET sensors, which have shown the selective ability to detect uranyl ions (UO_2_^+^) with detection limits approaching 10–100 nM in aqueous environments [55]. FRET efficiency is strongly distance-dependent (usually working over 1–10 nm) in nature and it strongly depends on the spectral overlap of the emission spectrum of the donor and the absorption spectrum of the acceptor. FRET is typically regulated by binding events with an analyte in COF-based sensing platforms through changes in donor–acceptor distance or orientation. This technology has been effectively used to identify pesticides, antibiotics and pharmaceutical residues. Interestingly, FRET-based sensors have the benefit of ratiometric sensing, which is where the ratio of the intensities of the two wavelengths of fluorescence is used to give the sensor automatic calibration to changes in the environment [56,57].

The two complementary enhancement mechanisms used to give the SERS effect are the electromagnetic and chemical ones. The mechanism by which the signal is amplified is the package of the powerful electromagnetic field in the proximity of localized surface plasmon resonance in noble metallic nanostructures (usually, Au or Ag). This is enhanced chemically by a fold or chemical amplification that occurs due to resonance in charge transfer processes during the process of analyte chemisorbence to the substrate surface. Fluorescence-based techniques include PET (which depends on modulating electron transfer) and FRET (which depends on distance-dependent energy transfer between donor–acceptor pairs), used to demonstrate turn-on/turn-off and ratiometric self-calibrating readings, respectively. SERS, in contrast, offers direct molecular-fingerprint information with plasmon-enhanced Raman scattering, with ultralow detection limits, and unambiguously recognizes analytes, but necessitates more complicated substrate preparation than the easier fluorescence-based techniques [58,59].

In spite of these fast innovations, the experimentation with COF-based biosensors on the commercial front is still an ongoing process. There are still difficulties in terms of large-scale, high-cost-effective production of high-quality COF thin films in the long-term stability of bio-immobilized species and in incorporating these materials into a microfluidic environment. This review will attempt to give a critical and thorough analysis of the prevailing circumstances of the state of the art of COF-based biosensing. We will discuss the core principles of design that apply to the synthesis of sensing-active COFs, and then we will discuss the various sensing mechanisms in application in detail. Lastly, we will discuss the most influential biomedical outcomes that have been reported in the past and provide insights into the upcoming challenges and opportunities that the next-generation platforms have in front of them.

## 2. Fundamental Design Principles of COFs

The reticular chemistry concepts that control the architecture of COFs have been applied in the construction of periodic and crystalline frameworks using organic molecule building blocks, covalently bonded and with great strength, to create structural cores. The main aim in designing COFs for biosensing is the desired fine-tuning of the pore geometry and surface functionality [62]. Strategies that designers use in exploiting symmetry include those based on the topology information of the precursors, including C_2_, C_3_ or C_4_ symmetric monomers, which determine the next lattice (e.g., hexagonal, tetragonal or Kagome). The primary focus of designs in two-dimensional COFs is a staggered or eclipsed layer of the sheets of aromatics, used to produce continuous one-dimensional nanochannels necessary to expedite diffusion of bi-analytes and facilitate complete signal transduction [63]. The biomedical use relies on the linkage chemistry adopted when designing the framework, hence the chemical stability of the framework. Although imine (C=N) and hydrazone bonds are preferred due to the high degree of crystallinity and easy formation, they are susceptible to hydrolysis in acidic biological environments [64,65,66]. The more modern concepts of design are oriented to the process of linkage conversion or developing an irreversible bond, e.g., a keto-enamine or thiazole bond, to preserve the design in the right structural state even in the long run. It may also be used to tune the interior milieu of the pore by the introduction of discrete functional groups by pre-synthetic or post-synthetic modification. A desired biomarker may interact with high-affinity using hydrogen bonds, π-π interaction or electrostatic interaction [67].

## 3. Synthesis Methodologies of COFs

COFs are produced by crystallization under equilibrium, and the delicate balance between the formation of bonds and error correction is necessary. These are the main ways that are applied to create high-quality COFs used in sensing and biomedical platforms. COFs can be synthesized by various synthesis methods and have specific reaction parameters for every type of them. The multi-synthetic COFs do not refer to a new structural class of COFs, but to designs and fabrication methods in which multiple synthetic strategies, such as solvothermal, interfacial, electrochemical, mechanochemical, etc., can be deliberately integrated to tune the structural, morphological, and functional properties of COFs for specific applications.

### 3.1. Solvothermal Synthesis

Solvothermal synthesis is the most active approach in the preparation of COFs since it is able to prepare highly crystalline materials with high surface areas. In this technique the solvents and the monomers are combined in a pressure-proof tube, typically a Pyrex tube, and the reaction undergoes a series of freeze–pump–thaw cycles. Maintained at controlled temperatures between 80 °C and 200 °C the reaction typically lasts several days (usually 2–9 days). Choosing the combination of solvents and the proportion of solvents is a critical decision because it plays a significant part in preceptor solubility, nucleation velocity, and the final porosity of the structure. Although this method is used to provide high-quality crystallinity, it has drawbacks in terms of long reaction times and it also uses closed tubes, which make this process difficult in large-scale manufacturing processes [68,69,70]. Dacheng Wei and their group highlight the solvothermal method as a prime method for synthesizing highly crystalline COFs. This report confirms that altering solvent composition and reaction temperature is crucial for controlling the material’s porosity and crystallinity [71].

### 3.2. Mechanochemical Synthesis

The alternative approach to COF preparation has become the mechanochemical preparation, which is praised because of its simplicity and environmental friendliness. This method requires the manual or machine grinding of precursors either in a mortar or in a mechanical mill at 290 °C; this process usually does not require the use of any organic solvents. The pressure needed in the process of grinding material is used to break and form new covalent connections within the solid precursors. The technique works especially well for generating COF nanosheets with thicknesses of 3–5 nm in less than a fraction of the time it takes for solvothermal techniques. Even though mechanochemically prepared COFs can sometimes have low surface areas relative to solvothermal prepared ones, the scalability and time-saving characteristic of the method make it a very appealing method for mass-producing sensing chips. To make the most out of it, it is recommended that microwave-assisted synthesis be utilized in preparing the products specified by Bell in the introductory section of the technical manual [72,73,74]. Also, a current report highlights mechanochemical synthesis as a fast, solvent-free route for COF synthesis. This work highlights that solid-state grinding can fabricate materials in minutes, a marked contrast to the multi-day procedure of conventional methods [75].

### 3.3. Microwave-Assisted Synthesis

Microwave-assisted synthesis uses microwave electromagnetic waves to excite the reaction solvent and cause the dipole molecules to move rapidly at high-frequency oscillations to create a uniform intramolecular frictional heat. By doing so, it offers a highly homogeneous condition of heating with the implication that the reaction time is significantly reduced. For example, 2D and 3D COFs with high levels of crystallinity can be easily obtained in 20 min, which is very short in comparison to the solvothermal method. In addition, the COFs prepared through microwaves of other frequencies tend to have similar surface area and porosity to those prepared by normal heating [76,77,78,79]. Xinle Li and their group confirm the productivity of the microwave-assisted method for rapidly fabricating crystalline COFs. This work emphasizes how this method achieves results analogous to those of traditional techniques but in a markedly reduced timeframe, often within minutes [80].

### 3.4. Sonochemical Synthesis

The sonochemical method can be used to synthesize COFs through the influence of extremely high-intensity ultrasonic waves of frequencies between 20 kHz and 10 MHz. The effect of these waves is the formation, growth and collapse of small bubbles that generate local energy and are used to speed up the formation of covalent combinations between the monomers. This higher value of power input might lead to the reaction times being much shorter; e.g., imine-bound COFs may have the synthesis process taking 60 min when operated under the aqueous condition. The crystal size of the sonochemically processed COFs is much smaller (~50 nm) than that of solvothermally produced ones, which can be advantageous in regard to the achievement of a uniform coating on the biosensing electrodes [81,82,83]. Reports on sonochemical synthesis illustrate its effectiveness for rapid COF production in green solvents. These studies focus on how ultrasonic irradiation facilitates rapid reaction kinetics and fabricates COFs for thin-film device applications [84].

### 3.5. Interfacial Synthesis

Interfacial synthesis is an expert method where the monomers polymerize at an interface between two immiscible phases, be it a liquid–liquid interface, gas–liquid interface, or solid–liquid interface. The technique is of critical importance especially in the production of large-scale highly ordered 2D COF thin films with well-controlled thicknesses. As an example, the controlled COF monolayers could be built through the aggregation of functional groups at the air–water surface. Due to confinement to one point of the reaction, one is able to produce morphologically homogeneous structures that are optimal for integrated electronic biosensing platforms where thin-film nanolayers can be needed to facilitate the best signal transduction [85,86,87]. Congjie Gao and their group highlight interfacial synthesis as a leading method for generating large-area, defect-free COF membranes. The work point outs the precise control this approach offers over film thickness and orientation, which is supreme for advanced separation and sensing applications [88].

### 3.6. Ionothermal Synthesis

In the ionothermal method, molten salts or ionic liquids are used as an ionic liquid and as a solvent in the synthesis of covalent structures. It is mainly employed to fabricate Covalent Triazine Frameworks (CTFs) and other Covalent Frameworks that are very robust, usually at high temperatures and pressures. Ionothermal synthesis provides significantly shorter reaction times than the traditional solvothermal technique and provides extraordinary stability to the resulting products in comparison. Indicatively, imide-based COFs have been made with molten ZnCl_2_ as a reaction medium, thus eliminating the use of other harmful organic solvents. Recent developments have shown ionothermal processes that can be carried out at ambient temperature and pressure, allowing the synthesis of three-dimensional COFs in minutes [89,90,91,92,93,94]. Bettina V. Lotsch and their group demonstrate the unique properties and advantages of imide-based COFs and, using this procedure, influence molten salts to play the role of both solvent and template, demonstrating the rapid production of highly stable, crystalline frameworks under comparatively mild conditions [90].

### 3.7. Photochemical and Radiation-Induced Synthesis

This method is intended to modify a substrate by designing photochemical and radiation-induced synthesis. The photochemical reaction utilizes ultraviolet (UV) or visible light and employs the light to catalyze the joining of the organic starting materials to a covalent bond. These processes are normally carried out at room temperature, so that there is no need to add high thermal input, hence making it more energy efficient. It is specifically applicable in the formation of morphologically homogeneous COF structures, particularly in cases where it leads to their quick formation. In the same manner, electron-beam- or gamma-radiation-induced polymerization was studied to enable the formation of COFs without any chemical catalysts in the catalyst-free pathway to high-purity materials [16,95,96]. Shuao Wang and their group illustrate the potential of this approach for generating grafted COF materials. These studies find that such techniques can adequately initiate reactions at ambient conditions, producing pure products without the requirement for high temperatures or additional catalysts [95].

### 3.8. Electrochemical Synthesis

Electrochemical fabrication enables the proper manipulation of reaction time and thickness of fabric at ambient conditions. It is specifically pronounced in direct coating with COF thin films on conductive surfaces in preparing the electrochemical biosensors. Relative to solvothermal protocols, which yield powders, electrochemical growth makes in situ growth of the framework upon the electrode surface feasible, which is critical to providing good electrical contact and mechanical adhesion, which are critical to attaining consistent signal transduction in gap generation platforms [17,97,98,99]. Shinsuke Inagi and their group highlight the technique’s precision and confirm that electrochemical synthesis gives unusual control over film morphology and crystallinity straight away on conducting reactants, which is necessary for developing advanced electrochemical devices [100].

### 3.9. On-Surface, In Situ Conversion

One of the ways through which an existing COF may be transformed into an alternate condition, influenced by the surroundings, temperature, pressure, radiation or the tiny few protons and metal ions, is in situ on-surface conversion. The above-mentioned variables have a high impact on the preparation of COFs with desired structures. This way enables both the acquisition of new capabilities of COF and the morphological homogeneity of the template. Through this, the size and uniformity of resultant COF nanolayers at the nanoscale may be regulated and, remarkably, through the application of a reaction on a single-layer-only system, which may be imposed by an applied electric field [39]. Yukui Zhang and their group report the in situ growth of olefin-linked COF nanofiber membranes via surface-mediated condensation, demonstrating this principle. This report illustrates how such transformation techniques can be harnessed to produce advanced, functional membranes with accurate structural control for targeted techniques like environmental remediation [101].

### 3.10. Overview of Synthesis Methodologies

**Figure 2:**
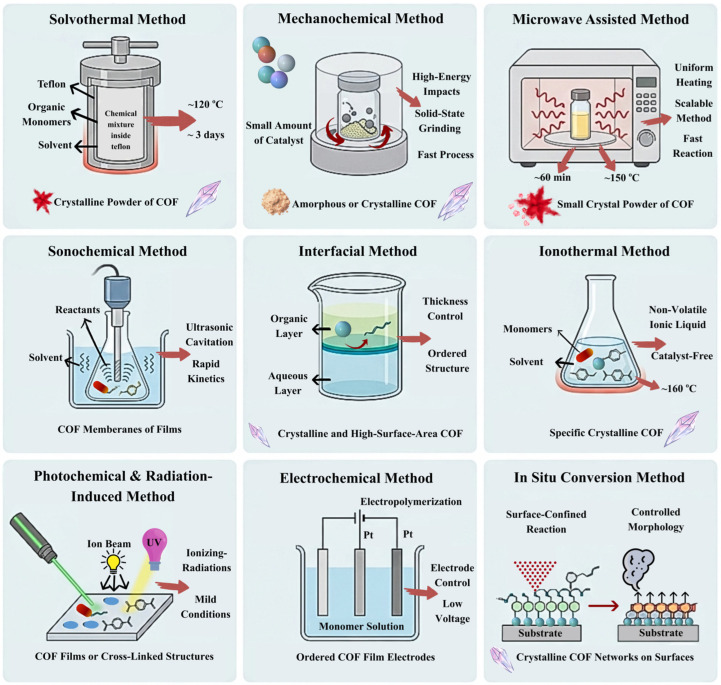
Overview of various synthesis methods of COFs.

The traditional solvothermal methods are also regarded as the most efficient methods to achieve high crystallinity and a defined pore structure, yet the trend is changing rather rapidly to on-surface and green processing in order to respond to clinical requirements. Microwave methods and mechanochemistry have demonstrated that it is possible to reduce days to a matter of minutes without disrupting the fundamental sensing properties of the framework by avoiding the time-consuming crystallization of the framework. It is required because of a strong need to develop COF-based platforms not just into lab-scale prototypes but scalable cost-effective biosensing chips under economical and environmentally friendly conditions. The overview of the major highlights of the discussed synthesis processes is presented in Figure 2 as well as in Table 1. Finally, the final transduction process of the biosensor continues to determine the choice of synthetic strategy to adopt. In situ electrochemical growth and interfacial synthesis is also the most promising approach to electrochemical and photoelectrochemical platforms since this alternative approach avoids the use of insulating binders and has better electronic contact between the COF and the transducer surface. According to the recent literature, the definition of the best synthesis in terms of surface area no longer depends on the capacity to be biocompatible and structurally stable in physiological conditions. It is the combination of any of these new advanced synthetic pathways that will eventually define the ability of COFs to compete with conventional materials in the next round of POC diagnostic equipment. The variety of synthetic methods mentioned above directly controls the crystallinity, porosity, level of defect, and distribution of functional groups of the final COFs. Key performance measures in sensing applications, e.g., sensitivity in detection, selectivity, response time, limit of detection, and important biomedical measurements—e.g., biocompatibility, drug loading capacity, release kinetics, and therapeutic efficacy—are determined by these physicochemical parameters. Therefore, to rationally design COF-based platforms with specific sensing and biomedical needs, it is crucial to know the synthesis–structure–performance relationship.

## 4. Sensing Applications of Covalent Organic Frameworks

### 4.1. Gas Sensing

Covalent organic frameworks become part of the vanguard of gas-sensing in the next generation due to their structural integrity and adjustable porosity. COF-based platforms, compared to traditional metal oxide sensors, which can be described as having a high operating temperature and sometimes mandate a high operating temperature, exploit a high-selectivity reaction in crystalline lattices of molecules to enable increased sensitivity and selective attributes in a room-temperature operating environment. These frameworks have been used differently to identify a wide variety of gaseous analytes, as explained in the subsequent sections.

#### 4.1.1. Alkaline Ammonia Vapor Detection

COFs provide a perfect platform for NH_3_ detection that is mainly based on Lewis acid and base interactions, and on hydrogen bonding networks [102]. The COFs such as TPE-Ph-COF (boronate-directed COFs) utilize the empty p orbitals of boron atoms that react with the extra pair electrons of NH_3_ molecules [103]. The interactions offer excellent quenching effects on the luminescence of the framework, thereby enabling detection of traces of ammonia in the organic solvents. The experimental results indicated that the low levels of ammonia, i.e., 1 ppm, had the capability of causing a 30% reduction in the intensity of the fluorescence, indicating the suitability of COFs for ultra-sensitive dosage detection of ammonia. In addition, more sensitive sensors have been invented that are based on resistance sensors using an imine-linked framework like TAPB-BPDA, whose lower detection limit is 10 ppb. The hydrogen bonding between the skeleton and the analyte is usually conducive to switching between these platforms, which effectively inhibits the perturbations of other commonly used gases, e.g., CO_2_ or H_2_ [104].

#### 4.1.2. Acidic Gases and Vapors

Acids such as HCl and trifluoroacetic acid (TFA) need to be identified to provide environmental protection and correct management of chemical procedures [105]. COFs involving imines and triazene-based COFs are mainly applied in this situation because the nitrogen nodes of these structures offer a great source of proton acceptors. The nitrogen centers’ arcs get easily protonated when they are treated with vapors of these acids, usually leading to a visible change in color. For example, Per-N-COFs change their color to dark brown with TFA exposure and exhibit an appreciable variation in UV-vis absorption bands. The wide range of these sensors is four orders of magnitude, with a limit of detection of 35 µmg/L. Also, triazine-based systems such as PBHP-TAPT-COFs would be capable of detecting HCl by colorimetric conversion (yellow to red), and electrical conductivity, where protonation would enhance the conductivity of the structure to up to 170-fold [104,106].

#### 4.1.3. Qualified Humidity and Water Vapor Detection

Humidity measurement is one of the crucial physiological measures, especially in wearable respiratory apparatuses that measure breathing rates. The use of COFs is very well adapted to this purpose since the orderly polygonal structure of the pores can be designed to selectively entrap water molecules using these interactions: keto-iminol tautomerism or hydrogen bonding. Water molecules in TAPP-DHNDA-COF nanofibers affect the tautomeric equilibrium, and the color will turn progressively from yellow to red when the relative humidity (R.H.) is increased between 20 and 100 percent [107]. Py-TT COFs are such thin-film structures that have a very good response time; i.e., the color change can be seen within 0.21 s and the recovery can be seen within 0.15 s. Thus, they are able to monitor human breath in real time [108]. Besides optical designs, impedance sensors also apply truxene-based boron ester COFs that rely on the Grotthuss chain reaction to facilitate the movement of protons and exhibit a sharp decrease in impedance in an extended R.H. range of 11% to 98% [109].

#### 4.1.4. Sensing of Volatile Organic Compounds (VOCs)

Volatile organic compounds are very dangerous to health in indoor settings, including benzene, toluene, and formaldehyde. COFs have a more favorable sensing platform for such non-polar molecules or weakly polar molecules due to the π—π stacking and electronic donor–acceptor interactions inherent to the framework. It is one of the most intriguing applications, as a BTA-TAPT-COF can be grown directly in direct contact with interdigitated electrodes (IDEs) to create a capacitive sensor of benzene. When the electron-saturated benzene gas is reacted with the electron-saturated triazine units at the substrate of the COF structure, it is observed that the capacitance typical of the device strongly increases. These kinds of devices have been shown to possess a limit of detection of 340 ppb and have an outstanding selectivity to benzene in comparison to other typical indoor gases like methane or propane [110].

#### 4.1.5. NO_2_ and Atmospheric Pollutants

Being a major cause of smog and respiratory diseases, nitrogen oxides necessitate high-performance sensors that can be used to monitor the quality of air in cities. Triazine 2D organic polymers have also been found to be highly applicable materials in NO_2_ sensing when they are added to IDE materials. Nevertheless, built through synthesizing mostly by catalytic cyclotrimerization, these scaffolds exhibit resistance-based NO_2_ sensitivities, and in principle can measure concentrations as low as 2.2 ppb. With a response and recovery time of less than 140 s, it becomes possible to monitor environmental pollution continuously [111]. Further, the applicability of TPB -DMTP-COFs in the detection of ozone (O_3_) has been recently pointed out; in the mechanism, ozone nucleophilically reacts with the imine bonds, creating nitro or aldehyde groups, or alternatively, creates a yellow-to-red color change due to the protonation of the compound with moisture [112].

#### 4.1.6. Toxic Sulphur-Containing Gas Detection

Hydrogen sulfide (H_2_S) is a highly toxic industrial product that has to be detected quickly. This has been achieved through the creation of luminescent nanotubes such as PNT-1 that are developed as a result of condensing triazine derivatives and bipyridine to give a fluorescent structure. The fluorescence of these nanotubes can be quenched by S_2_^−^ ions or gaseous H_2_S. This has resulted in the development of wet-based test papers which can be dipped into the suspension of COFs, and when the paper is exposed to an H_2_S environment, the fluorescence of the paper is extinguished instantly and this provides a cheap, portable and very visible system of detection of this dangerous gas [113].

#### 4.1.7. Greenhouse Gas Measuring and Capture of Carbon

Methane (CH_4_) and carbon dioxide (CO_2_) detection is an emerging field of COFs as there is a transitional inequality between gas storage and analyte analysis. The great affinity of some COFs to CO_2_ is usually made possible by the introduction of polar functional groups like amines or hydroxyls in the walls of the pore interior. On adsorbing CO_2_ molecules, it alters either the refractive index or capacitance of the framework, which can be used as a transduction channel in a greenhouse gas concentration detector. In the case of methane, which does not have a permanent dipole moment, detection involves the definitive confinement of the pore size to the kinetic diameter of the molecule, where the host molecules and guests interact in the confined cavity to change the vibrational resonances of the skeleton of the COF that are observed through surface-enhanced Raman spectroscopy (SERS) or infrared spectroscopy [114,115,116].

#### 4.1.8. New Breathalyzer Technology to Detect VOCs

In the case of the most evident illustration of NextGen biosensing, COFs are an example that has been used in the form of medical breathalyzers and are used to detect endogenous volatile organic compounds (VOCs). COFs can be functionalized specifically to capture a certain marker, such as acetone to track ketosis, as well as nitric oxide in order to track airway inflammation with very high specificity. The electrochemical current is sensitive to catalytic oxidation of these VOCs leaving marks on the COF sheets in terms of noble metal nanoparticles or to alteration of the functioning of a field-effect transistor (FET). The specified methodology offers a non-invasive approach to diagnosing this problem, substituting blood testing with a simplistic breath examination, an instance of shaping COFs from industrial tools into inseparable elements of contemporary diagnostic tools involved in the field of biomedical practice [117].

### 4.2. Inorganic Ion Sensing

#### 4.2.1. H^+^ (pH) Sensing

Recently, the monitoring of pH has been reported with high specificity by replacing crude solution-based assays with biocompatible frameworks. A study by C. L. Zhang and collaborators showed that poly-D-Lysine (PDL) functionalization of 2D COF nanosheets addresses the drawback of the platform having intrinsic hydrophobicity and allows increased solubility in aqueous dispersions, biocompatibility and endocytosis performance due to the merits of poly-D-Lysine functionalization [118]. The principle of the method is the reversible protonation and deprotonation of the weak basic sites, nitrogen atoms in the COF structure. When acidic conditions (pH < 4.5) are present, the nitrogen atoms are protonated which results in an increase in fluorescence but with blue-shifted fluorescence at 428 nm. Conversely, deprotonation in basic conditions (pH 9–13) causes gradual elimination of the fluorescence. The reversibility between acidic/neutral and basic conditions of this fluorescent pH sensor has been very good as the crystallinity of the sensor has been able to sustain high measurements of crystallinity during repeated processes. The first approach to be evolved was the COF-JLU4 platform, which was developed as a COF-based fluorescent pH sensor and can broaden the scope of COF uses in environmental and biological pH biosensing. The authors then demonstrated the versatility of PDL-modified COFs in two platforms, in vivo in zebrafish models of pH mapping, and in vitro imaging of cancer cells without a control group, which represents the breaking point of the so-called smart bio-imaging probes. The COF-JLU4 platform was among the first approaches to be developed as a COF-based fluorescent pH sensor, which increases the range of COF applications to environmental and biological pH biosensing.

#### 4.2.2. Metal Ion Sensing

Essential transition metals such as Fe^2+^, Mg^2+^ and Zn^2+^ are fundamental to the physiology because the sensing of the Fe^2+^/Fe^3+^ ratio is a basic requirement for anemia diagnosis and management. The anthropogenic release of heavy metal ions into the environment poses a terrible threat to the health of beings. These ions are very toxic and are capable of bioaccumulating, which results in irreversible renal, neurological and systemic damage. The reason why COFs are considered an elite class of sensing materials is their high porosity, quick analyte diffusion, and designable structures, which allow the chelating ligands to be installed flawlessly. The researchers have created the emerging COFs by changing the steric and the electronic space of the crystalline COFs, which are highly selective and sensitive to the different array of metal ions [119].

#### 4.2.3. Hg^2+^ Sensing

Mercury has a huge historical impact through Minamata disease and that is why it is also one of the most examined toxic metals. A COF that is functionalized to detect Hg^2+^ is often sensitive to the soft Lewis base sites, such as thioether groups of sulfur-containing thiol groups. W. Wang et al. reported the preparation of a COF-LZU8 that had a hexagonal pore structure which was specially designed to sense mercury. The translation of a COF to Hg^2+^ ions using electronic transformations led to a linear time-dependent decrease in fluorescence and an LOD of 25 ppb in acetonitrile matrix, which is superior to many thioether functionalized chemosensors. Interestingly, the uptake capacity of this COF was high and this feature allowed it to act as a removal agent at the same time, whose regeneration requires Na_2_S solution, which is readily available [120]. J.D. Qiu et al. applied a carbohydrazide-based flexible linkage on the pyrene-based structure to create TFPpy-CHYD. The CHYD units were used in sorting Hg^2+^ and were able to achieve the highest levels of adsorption of 758 mg/g and an LOD of 17 nM (with DMF as co-solvent) [121]. AuNPs in a Tp-Bpy composite (bipyridine-based COF) by J.D. Qiu et al. was another approach, in which the COF was a substrate on which gold grew in situ. The presence of Hg^2+^ could be determined by the development of a coating of gold-amalgam layer which oxidized TMB to a bright blue state and enabled the detection of mercury even at a low concentration of 0.33 nM, and the applications for tap water, Ganjiang river water, and Poyang lake water were also tested, with recovery rates of Hg^2+^ close to 100% [122].

#### 4.2.4. Cu^2+^ Sensing

Copper is not as toxic as mercury but its presence in the environment should be monitored. Typically, the azine- or triazine-based COFs are applicable to detecting Cu^2+^ and they are able to chelate the transition metal. As an example, X. Liu et al. presented an azine-conjugated COF-JLU3, which was stabilized by large tert-butyl groups. These consisted of its nitrogen atoms and hydroxyl groups as receptors, which resulted in a considerable reduction in the fluorescence, with an LOD of 0.31 µM, and the sensing was performed in an organic solvent (THF) [123]. Y. Xiong et al. synthesized a Covalent Triazine Framework (CTF) as a colorimetric sensory framework. The CTF/Cu^2+^ had an outstanding peroxidase-like activity and catalyzed the breakdown of H_2_O_2_. The findings proved the colorimetric investigation of Cu^2+^ with a low LOD of 0.05 µg/L. This colorimetric test was successfully validated in food samples such as eggplant and Chinese water chestnut with around 100% recoveries [124]. D. Jiang et al. proposed the use of carbon conjugate COFs that contained cyano-functional groups. These compounds were very chemically stable under harsh conditions (12 M HCl, 14 M NaOH, and 1-year air exposure) and, in addition, offered a very sensitive fluorescence-off signal in the presence of Cu^2+^ and have been detected at 88ppb [125].

#### 4.2.5. (UO_2_)^2+^, Precious and Transition Metal Targets

The nuclear energy industry requires extremely selective Uranium (UO_2_)^2+^ sensors and mining. Therefore, the functionalized versions of the COFs with actinide-binding groups can be viable materials in that case. J.D. Qiu et al. synthesized a fluorescent “TFPT-BTAN-AO” framework that is capable of (UO_2_)^2+^ capture with an uptake capacity of 427 mg/g. As Uranium is adsorbed, the fluorescence emission of TFPT-BTAN-AO decreases linearly, providing the LOD of 6.7 nM in aqueous solution of pH 6.0 that is necessary for sensing the trace leakages in nuclear facilities [126,127]. COFs are also promising candidates for the sensing and recovery of precious metals, such as gold, palladium, nickel, etc. Yu et al. synthesized a thioether-functionalized TTB-COF for sensing Au^+^, which has an LOD of 0.87 mM in acetonitrile (1.39 μM in water) and a 560 mg/g recovery capacity [128]. Y. Lu et al. reported PY-SE-COF for fluorescence sensing and removal of Pd^2+^. The framework exhibited high chemical stability and high selectivity for Pd^2+^, and a 0.45 μM detection limit, while the removal efficiency remained above 95% even after 10 consecutive cycles [129]. A bipyridine-based COF for transition metal detection was reported by Q.L. Deng et al., and the achieved LOD of Ni^2+^ at pH 10 was 68 pM [130]. Furthermore, the detection of chromium (Cr^3+^) has been advanced through the use of DNA-functionalized COF hybrids. M. Du et al. developed an electrochemical biosensor by coating a glass carbon electrode with a DNA/CoPc–PT–COF@Cu–MOF composite [131].

#### 4.2.6. Pb^2+^ Sensing

Lead is a chronic toxic metal compound that accumulates along the food chain and adversely impacts the hepatic, nervous, and cardiovascular systems; therefore, sensitive and quick Pb^2+^ detection is a matter of concern. Pb^2+^ sensing by COF models has mainly relied on electrochemical and photoelectrochemical (PEC) signal transduction. Wang and colleagues reported a TAPB-DMTP-COF/CPE sensor by modifying a gold electrode with a COF-containing amino group (TAPB-DMTP-COF) which led to a TAPB-DMTP-COF/CPE sensor of Pb^2+^. Amino groups of TAPB–DMTP-COFs allow easy accumulation of Pb^2+^ and the differential-pulse anodic-stripping voltammetry had a linear response of 0.005 to 2.0 μmol/L and an LOD of 0.0019 nmol/L [132]. It was a further improvement of the stated PEC on–off–on Pb^2+^ sensor as developed by Zhang and co-workers, who made use of a porphyrin-based TAPP-COF thin film as a photocathode on PET-ITO. The porphyrin units and ordered channels of COF are helpful in the transportation of the charge as well as an effective light harvester and the CdSe@SiO_2_ quantum dots, with a chain reaction of hybridization by the structure, act as the quenchers that should initially shut the photocurrent. The CdSe@SiO_2_ quantum dots on the COF surface are selectively removed by Target Pb^2+^ ions, which recovers the photocurrent and quantitative measurement of 0.05–1000 nM with a very low LOD of 0.012 nM. The sensor demonstrated perfect selectivity and was validated in tap and lake water samples with around 100% recoveries [133].

#### 4.2.7. Fe^3+^ Sensing

Oxygen gets transported by iron and other redox enzymes, and therefore, abnormal amounts of Fe^3+^ are a grave health risk, particularly on biological and food surfaces. COFs offer π-conjugated and highly porous scaffolds that are rigid and offer high levels of coordination positions and strong fluorescence to coordinate with Fe^3+^. Early investigations by Yang et al. showed two porous COFs derived through polyimide, which emit strongly through 0-transitions of their highly delocalized, rigid backbones, PI-COF-201 and PI-COF-202 [134]. When Fe^3+^ is coordinated into the pores, the COFs exhibit an efficient quenching of fluorescence since the energy of the excited state of the framework is transferred to the free d orbitals of Fe^3+^, which allows quantitative detection between 0.005 and 0.4 mM with LODs of about 0.13 μM and 0.22 μM with PI-COF-201 and PI-COF-202, respectively, using PI-COF suspensions mixed with water.

Later designs, especially hydrazone-versatile and wall-functionalized COFs with O, N and O chelating groups, also improved the sensitivity and selectivity towards Fe^3+^. Common applications reached LOD values of 64 nM in ethanol, and most of these fluorescent COFs can also detect other dangerous oxyanions and metal ions (such as CrO_4_^−^, Cr_2_O_7_^−^, MnO_4_^−^, etc.), also illustrating the multivariable property of these sensors.

The group of Zhang et al. reported a hydrazone-linked Tfpa-Mth COF, which can be easily applied as a fluorescent probe as well as the mass-sensitive coating of quartz crystal microbalance (QCM) chips. The Tfpa-Mth COF thin film, when uniformly produced on the amino-modified QCM electrode, which is grown in situ, is able to hold the Fe^3+^ of the solution, and hence, the resulting resonant frequency change can be detected, thus enabling real-time tracking with high selectivity. Compared to fluorescence quenching in solution, the COF-coated QCM platform allows real-time, label-free detection of low concentrations of Fe^3+^ in complex samples [135]. Moreover, some of these COFs have been used for sensing, are mentioned in Table 2 highlighting their names, bonding types and specific results to the applications.

## 5. Biomedical Applications of COFs

Serious diseases, especially cancer, cannot rely only on early detection but also require precise and accurate targeted therapies. COFs have unique properties, including high surface area, chemical versatility, and tunable pore sizes, etc., and thus, they are emerging and promising platforms for therapeutic applications. Unlike many inorganic or metal–organic carriers, COFs are composed of lighter elements such as C, O, N, B, etc., which removes the concerns of heavy metal toxicity. COFs are usable for multifunctional treatments with controlled drug release and high specificity. The stability and functionalization potential of COFs support their usage in various therapies such as photothermal therapy (PTT), sonodynamic therapy (SDT), drug delivery, photodynamic therapy (PDT), immunotherapy, etc. This section will explore therapeutic applications of COFs, mainly focusing on PTT, PDT, drug delivery, and combined therapy.

### 5.1. Photothermal Therapy (PTT)

Photothermal therapy (PTT) is a novel treatment modality that is based on the utilization of external light sources, especially lasers, to target the generation of thermal damage in tumor cells, which eventually results in the destruction of tumorous tissue. However, unlike PDT, PTT is oxygen-independent and it can be considered a promising therapeutic option in hypoxic tumors. However, the use of PTT has not been extensively adopted in the clinical industry due to the possibility that lasers of high intensity will ultimately destroy normal cells or tissues. The potential of photothermal conversion agents (PTAs) with such phenomenal ability to transform light energy into thermal energy has a huge potential in minimizing the amount of light power that is needed, thereby eliminating potential damage to the surrounding normal tissues [140,141]. Still, low biocompatibility and low photostability, inability to target tumors, and clearance impediments pose a large adverse effect on the translation of the targeted intra-tumoral distribution of the PTAs to efficient tumor therapy in clinical practice.

The large surface area and tunability of the porosity of COFs enable them to accommodate and deliver high amounts of PTAs and are further enhanced by the stability of the covalent bonds forming inside the COF structures. In addition, the facile functionalization with targeting factors makes COFs more tumor-selective, decreasing off-target effects and minimizing the effects on normal tissue. As an example, COFs loaded with heteropoly blue (HPB), which is an optimal PTA, have been synthesized using a one-pot method and broken down to release HPB in the acidic tumor microenvironment, thereby causing direct PTT by using the 808 nm NIR laser irradiation [142]. The versatility of COFs and the possibility of including other therapeutic agents also provide opportunities for combined therapies in cancer and might enhance the treatment efficacy.

### 5.2. Photodynamic Therapy (PDT)

The mode of action of photodynamic therapy (PDT) is based on the presence of a low-toxicity or non-toxic photosensitizer that concentrates at the tumor site. The PS produces high levels of reactive oxygen species in response to an external light source of the right wavelength, resulting in the successful targeting and killing of tumor cells. PDT has become a potential treatment modality in clinical cancer therapy, taking into consideration its capability of exactly targeting localized cancer cells, which would allow the treatment to counteract the side effects that are usually presented by chemotherapy treatment [143]. COFs can be functionalized, the surface can be tailored, and the pore size can be adjusted, with PSs being able to be included in the scaffold, and thus increase accumulation in tumor tissues, because the surface area is significant, and thus, can be functionalized. Further, COFs can be functionalized with care to preferentially target tumor locations to avoid immune response and on-demand delivery of PSs to particular locations in response to stimuli, which increases PSs’ functionality. A PDA-coated COF 1 material immobilizing NIR dye indocyanine green (ICG) has been proposed and is indicated to possess superior ROS generation capability, which is stronger than the conventional PDT in hypoxic tumors. The prepared ICG@COF-1@PDA was found to exhibit outstanding PDT therapeutic effect and resulted in colorectal cancer and murine breast cancer metastasis inhibition [144]. Also, the structural tunability and large surface area of COFs provide an opportunity to use them as PSs because photoactive components (porphyrins or acridines) can be incorporated into them, which will be able to absorb light successfully and generate ROS under photoactivation. Guan et al. were one of the first to propose the use of COFs as novel PSs in 2019 on a platform of benzene-1,3,5-tricarboxaldehyde and tert-butyl 4-aminophenyl carbamate, in which they had synthesized a novel BODIPy-modified COF by a classic solvothermal reaction. The resulting COF showed an outstanding capability to induce an increased PDT activity and increased anticancer activity that displayed substantial growth inhibitions of HeLa and MCF-7 cancer cells under green light but had little growth-inhibitory effect on the MCF-10A normal cells. Further, PEG-modified renal-cleavable ultrasmall porphyrin-based COF nanodots have been reported to be well-stabilized and biocompatible. The resulting COF nanodot–PEG induced the generation of ROS by light, leading to the enhancement of PDT performance on cancerous cells [145].

### 5.3. Drug Delivery

**Figure 3:**
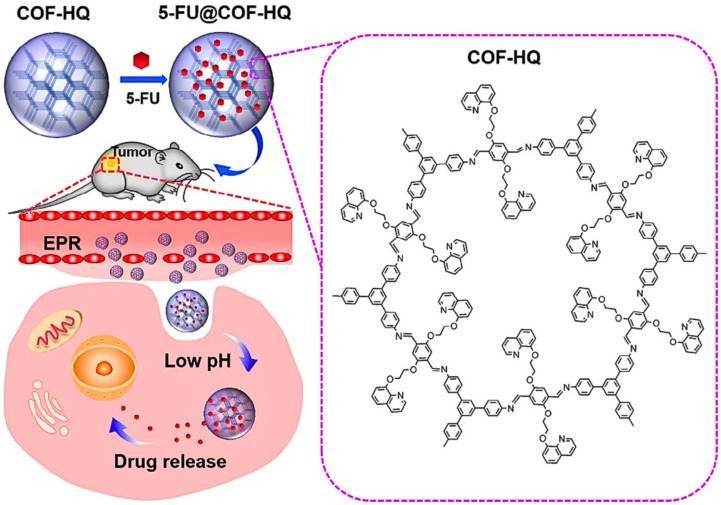
Schematic of (COF)-HQ for drug delivery and targeted release in vivo. Adapted from [151].

Many chemotherapy medications, including doxorubicin (DOX), have been identified to be poorly tumor-targeted and released statistically without any control, resulting in severe side effects. The unique characteristics of the COFs including modular porosity, configurable pore morphology, and a high surface area render them an ideal drug delivery carrier. Weak interactions, e.g., hydrogen bonds, electrostatic interactions and van der Waals interactions with COF functional groups, can be used to ensure that drug molecules are firmly lodged into the correct pores of the COF [146]. Further, good biocompatibility and the possibility of surface modification enhance tumor targeting and promote successful drug delivery in disease tissues/cells and reduce off-target effects as well as system toxicity [147]. Initial attempts to use COFs as a drug carrier were reported in 2015, with Yan et al. [148] being able to initiate the synthesis of a three-dimensional, porous, imide crystal variant of the COF (PI-COF) and entrap ibuprofen with a high loading factor of 20 wt%, demonstrating a controlled release rate of about 95 percent over approximately 6 d. Since that time more and more COFs have been used in drug delivery applications. The second study was able to confirm that drug-loaded COFs have the potential to be used in treating cancer. Pirfenidone (PFD), an antifibrotic agent, was loaded into the imino-based COFs and subsequently functionalized with poly (lactic–glycolic acid) copolymer–polyethylene glycol (PLGA-PEG), which can accumulate and release PFD at the tumor site following the intravenous injection [149]. The liberated PFD causes downregulation of the tumor extracellular matrix (ECM), including hyaluronic acid (HA) and collagen I, which significantly decreases the solid stress of the tumor and increases photodynamic therapy (PDT) of the tumor in vivo. In addition to this, when drugs are administered, preterm drug spillage can interfere with the intentional release of drugs at the chosen location, resulting in significant systemic toxicity, multidrug tolerance and therapy failure. To overcome the shortcomings of conventional drugs, which include tissue accumulation, severe toxicity, and decreased therapeutic efficacy, COFs can be drug-loaded and designed to release the drug where required in diseased tissue, e.g., the tumor microenvironment [150]. Mehvari et al. highlighted COFs for their well-arranged porous structure, excellent binding sites, high chemical and thermal stability, large surface area, and low density. The authors pointed out that the ultra-porous nature of COFs enables them to have a sufficient drug-loading capacity in the DDS, and that the absence of metals in COFs may be able to keep the potential toxicity at bay entirely, as long as it involves more biocompatibility in the DDS. The targeted drug delivery with 5-FU@COF-HQ is presented in Figure 3. With respect to drug release profiles, the review reports that pyridine-based COFs are explicitly characterized as having a notably sustained system where a huge number of drug molecules were released over a standard time (4 days), but fluorine-functionalized COFs could be second with respect to the release of drugs over 3 days. Importantly, redox-responsive COFs have an almost 90.00% release of DOX in 2 days, whereas amino-functionalized frameworks have released a similar amount of drug in 5 days. The authors also emphasize that biodegradable COFs may be of the utmost interest, such that the resulting COFs remain stable, dispersible, flexible, and targetable after use, and indicate that coating COFs with biopolymers or altering their surface characteristics might solve this issue. Notably, to achieve performing cellular absorption in in vivo drug delivery, the best size of the particle ought to be 200 nm or less [151]. So, the COFs have been found to have good encapsulation and drug release, as a result of their metal-ion-free architecture and high biocompatibility, making them next-generation assemblies for targeted cancer therapy.

### 5.4. Combined Therapy

As versatile drug carriers, COFs can readily integrate a range of therapeutic agents, such as anticancer drugs, PSs, and PTAs, in their porous frameworks, therefore allowing PDT and/or PTT to be combined with other therapeutic agents, and particularly with chemotherapy (CT), as therapies. When the agents are introduced to the tumor to take advantage of the simultaneous activity of PTT and CT, these agents are either prompted to action in response to external (e.g., light) or internal tumor microenvironment (e.g., pH, hypoxia) factors. The effectiveness of either of the different therapies could be increased by this approach with the aim of eliminating tumors completely. A variant of a nanoscale COF is a carrier for a porphyrin-based PS via the use of covalent grafting and a naphthylamine-based PTA via noncovalent linkages, which improves the photostability of the PS and the photothermal conversion ability of PTA since the extended pi-conjugated structure contributes immensely to the production of ROS under both visible light and NIR light at 808 nm that inhibits the proliferation and systemic metastasis of MCF-7 breast cancer cells. PDT and PTT, when used together under light illumination under 630 nm, resulted in total inhibition of the tumor growth in the mice bearing tumors of 4T1. Moreover, long p-conjugated systems of COFs could be useful PTAs themselves.

**Figure 4:**
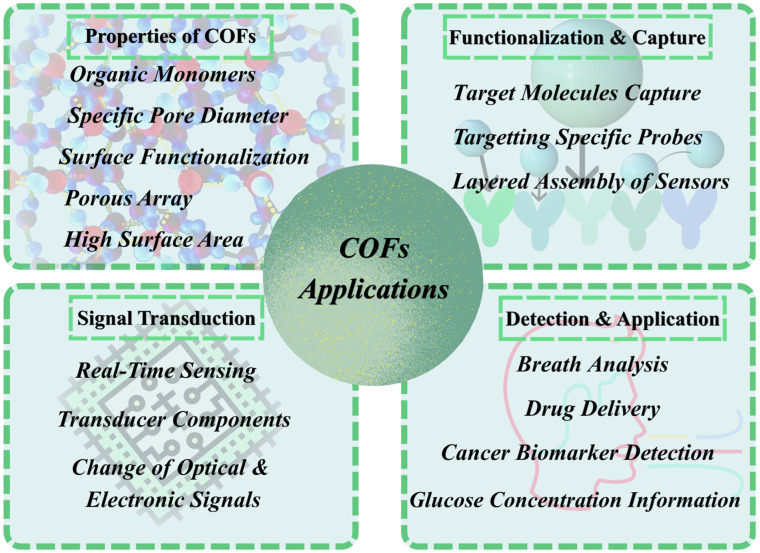
General illustration of COFs’ applications.

An example of such is donor–acceptor COFs that normally consist of an electron-rich component and an electron-deficient component, which cause the system to rapidly absorb, especially in the NIR range, and are an ideal PTA candidate for penetrating deep tissue and effective photothermal conversion [152]. In addition to that, it is better to use PTT at low or mild temperatures to minimize the damage to normal cells. To achieve the heat resistance of the cancer cells, a nanoscale photothermal COF was loaded with the gambogic acid (GA), an inhibitor of heat shock protein 90 (HSP90). Therefore, in cancer PTT at low temperatures, under the COF-GA nanoagent treatment, this strategy greatly decreased tumor growth, which has high potential. The fact that enormous gains can be acquired with the aid of COFs in cancer treatment, such as the possibility of targeted delivery of a drug, better therapeutic capacity, reduced adverse effects and the possibility of avoiding the instability of a particular drug, will have to be met by the fact that problems like complexity in design, possibility of regulating the release of more than one drug, and high price, influence the ability to scale up the complex [153]. The major properties of COFs along with their general sensing and biomedical applications have been elaborated in Figure 4. Moreover, various COFs have been mentioned in Table 3 highlighting their names, biomedical applications and specific results.

## 6. Challenges and Future Perspectives

**Figure 5:**
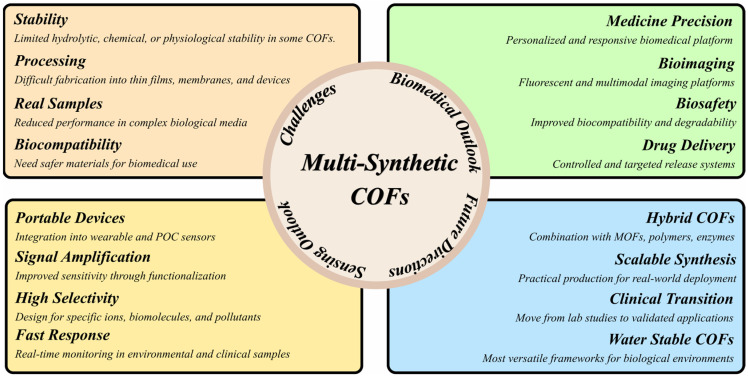
Challenges and future perspectives for multi-synthetic COFs.

Although the advances made in COF-based sensors are incredible, there are a number of severe challenges that hinder the conversion of these prototypes into a real-life set-up. One of the inherent disadvantages is that a large number of COFs have low hydrolytic/chemical/physiological stability, limiting their long-term performance under wet and biological conditions. The result of this instability is directly related to their ability or incapacity to be reused or to be used in continuous monitoring situations. The challenges, various sensing and biomedical aspects, and future perspectives are elaborated in Figure 5. Moreover, the challenge of producing COFs in thin-film impressions, membranes and portable-device formats is a significant bottleneck to practical use. Even the most sensitive COF sensors will not be able to go beyond the laboratory to the industrial level without scalable and reproducible processing methods. With complex biological environments, COF-based sensors are prone to poor performance caused by biofouling, nonspecific adsorption, and coexisting biomolecules, so there is a desire to develop antifouling surface approaches and matrix-resistant designs.

In the future, the field needs to focus on creating water-stable COFs that are highly biocompatible and have a well-defined degradability to be used in biomedicine. The introduction of COFs in wearable and POC devices, achieved via signal amplification principles and making hybrids (COFs/MOFs, polymers, or enzymes), includes a new layer of research. The great selectivity of certain ions, biomolecules, and pollutants and the short response times needed to enable real-time monitoring are major design objectives. Finally, scalable synthesis, strict biosafety evaluation, and personalized medicine-focused responsive platforms will be necessary to achieve the successful clinical translation of COF-based platforms. In this way, COF sensors can prove their potential as revolutionary environmental monitoring, disease diagnostics, and precision therapeutics tools.

## 7. Conclusions

Covalent organic frameworks are highly porous structures created through the covalent bonding of organic components. The ability to control precisely the porosity, surface area, functionality, and structure of the COFs offers a promising opportunity for many applications including sensing and biomedical ones. The wide range of synthetic methodologies varying from solvothermal, mechanochemical, microwave, sonochemical, interfacial, ionothermal, photochemical, and radiation-assisted techniques to electrochemical and surface-mediated approaches demonstrates the variable nature of COFs, facilitating precise control of porosity, crystallinity, and functional sites. Moreover, COFs highlight remarkable execution in sensing applications, incorporating gas detection and inorganic ion sensing, because of their high surface area, variable pore environments, and inherent chemical stability. In biomedical circumstances, COFs offer a multifunctional scaffold for photothermal therapy, photodynamic therapy, drug delivery, and merged therapeutic techniques, emphasizing their potential in next-generation therapeutic treatment.

Advanced methods of sensing like FRET, PET, or SERS, are being used in contemporary COF-based biosensing to increase the sensitivity of the measurements. Employing such methods allows detecting target molecules in very low concentrations, which are crucial for early diagnostics of the disease, where biomarkers usually can be found in biological systems. Moreover, some linkages like imines and keto-enamines in the COF structure guarantee chemical stability under extreme acidic and basic conditions. Being good substrates for synthesis, COFs allow selective immobilization of various elements like aptamers, fluorophores, gold nanoparticles, and enzymes, making possible the realization of different functions such as molecular sieving, signaling amplification, reducing LOD, and concentration of analytes. Advanced sensing systems based on COFs make it possible to identify cancer cells, volatile organic compounds in human breath, and blood glucose for diabetics, among other things.

By compiling expertise across synthesis, structural design, and applications, this review gives a clear insight into how COFs can be tuned for particular functional outcomes. The insights on multi-synthetic techniques with application-based design not only provide basic knowledge but also show the way for practical applications in healthcare, environmental monitoring, and analytical sciences. Collectively, the highlights covered in the review demonstrate the transformative potential of COFs as versatile, high-performance materials, strengthening their crucial role spanning fundamental chemistry and real-world technological resolutions. The pace of progress in the discussed areas is impressive; however, commercialization of COF-based sensors is still in its infancy. The main problems relate to the low price, manufacturing high-quality products, scale-up, and stability. Incorporation of such crystal elements into microfluidic systems is required for creating portable decentralized point-of-care devices. The future of COFs lies in reaching ultrahigh sensitivity and selectivity for analytical instruments used in clinical diagnostics and environmental safety.

## Figures and Tables

**Table 1:** Overview of main features of COF synthesis methodologies.

Methods	Principles	Advantages	Disadvantages
Solvothermal	Heat and dissolve monomers in organic solvent	High crystallinity, versatility, well-defined structures	High energy needs, complexities, secondary compound formation
Mechanochemical	Grind monomers to induce chemical reactions without solvent	Room temperature, solvent-free method, environmentally friendly	Less control over porosity, low crystallinity
Microwave Assisted	Heat reaction mixtures using microwave radiations	Uniform temperature control, higher efficiencies	Scaling up challenges, limited compatibility, special microwave equipment needed
Sonochemical	Heating of reaction mixtures using ultrasonic waves	Cost-efficient, lower reaction times	Scaling up challenges, low crystallinity, specialized ultrasonic equipment needed
Interfacial Synthesis	Polymerization at interface between two phases	Controlled thickness, fabrication of larger area 2D COFs	Precise control of reaction conditions is needed, time-consuming
Ionothermal	Heating monomers in molten salts or ionic liquids at high temperatures	High stability, reduced reaction times, reduced usage of harmful organic solvents	High costs, high temperature and pressure are needed
Photochemical	Activation of monomers for COF formation using light irradiation	Temporal and spatial control of reaction, mild synthesis conditions	Specific wavelength of light needed, limited photostability of monomers
Electrochemical	Polymerization of monomers by applying electric fields	Ease in reaction control and monitoring, mild synthesis conditions	Low crystallinity and purity, special electrochemical equipment needed
In Situ Conversion	Conversion of bonding of existing COF types to make new COFs	Ease in monitoring the dynamic covalent linking, uniformity in morphology	Complexity of operation, requires existing COFs.

**Table 2:** Literature review of COF-based sensing.

Analyte	COF	Detectable Signal	Detectable Range	LOD	Specific Binding Site	Ref.
NH_3_	HMP-TAPB-1	Conductivity	1–200 ppm	1 ppm	Heptazine	[136]
	COP-1	Fluorescence (turn on)	-	5.89 × 10^−4^ mL/mL	Triazine	[105]
	TAPB-BPDA	Conductivity	5–100 ppm	10 ppb	Imine	[137]
	COF-DC-8	Conductivity	2–80 ppm	56.8–70 ppb	-	[104]
H_2_S	COF-DC-8	Conductivity	2–80 ppm	121 ppb	-	[138]
	PNT-1	Fluorescence (turn off)	-	53 ppb	Triazine, pyridine	[113]
H_2_O	Py-TT	Chromism	0.64–0.98 p/p_o_	-	-	[108]
	DUT-175	Chromism	33–94% RH	-	Imine	[139]
	COF-TXDBA	Conductivity	11–98% RH	-	Boronate	[109]
	TAPP-DHNDA	Chromism	20–100% RH	-	Iminol	[107]
HCl	COP-1	Fluorescence (turn off)	-	1.096 × 10^−4^ mL/mL	Triazine	[105]
	BCTB-BCTA	Fluorescence (turn off)	1–25 mM	10 nM	Imine	[66]
	PBHP-TAPT	Chromism	20–3000 ppm	20 ppm	Triazine	[106]
Benzene	BTA-TAPT	Capacitance	500 ppb–100 ppm	340 ppb	Aromatic group	[110]
O_3_	P-COFTPB-DMTP-COF	Chromism	-	0.1 ppm	Imine	[112]
TFA	Per-N-COF	Chromism	0.035–110 mg/L	35 µg/L	Imine	[65]

**Table 3:** Applications of COFs for various disease therapies.

COF	Application	Target	Role of COFs	Ref.
PLGA-PEG	Drug delivery	CT26-tumor-bearing mice	Loading and target release of pirfenidone at tumor	[149]
Cy@COF-1	Combined therapy	Cancer cells (HeLa)	Loading of NIR dye cypate and ^1^O_2_	[154]
Fe_2_O_3_@COF	SDT	4T1 tumor-bearing mice	PTA	[155]
HPB-loaded COF	PTT	Cancer cells	HPB carrier for controlled release and enhanced biocompatibility	[142]
Porphyrin-COF	SDT	4T1 tumor-bearing mice	Using ultrasonic irradiations, produced singlet oxygen	[156]
COF-909-Ni	Immunotherapy	Cancer cells (4T1)	Pyroptosis induction	[157]
F68@SS-COF	Drug delivery	Cancer cells (HepG2)	Loading and delivery of DOX	[158]
BODIPY-modified COF	PDT	Cancer cells (HeLa & MCF-7)	PS for improved PDT efficiency	[159]

## Data Availability

No new data were created or analyzed in this study.
